# Tennessee Veterinary Medical Association

**Published:** 1897-11

**Authors:** Joseph Plaskett

**Affiliations:** Secretary


Secretary.

### TENNESSEE VETERINARY MEDICAL ASSOCIATION.

THE second annual meeting was held at the Tulane Hotel, Nashville, Tenn., on Monday, September 6th. In the absence of the President, the first Vice-President, Dr. Joseph M. Good, called the meeting to order. The minutes of the last meeting were read and approved. The Committee on Permanent Organization reported that they had drawn up a Constitution and By-laws for the approval of the Association. The Secretary was then instructed to read the proposed Constitution and By-laws, which was done, and after some modifications and alterations they were adopted.

The regular order of business was then taken up. On roll-call the following members answered to their names: Dr. Good, Chattanooga; Dr. White, Chapel Hill; Dr. Rauch, Memphis; and Drs. Hagyard, Rayen, Scott, and Plaskett, of Nashville. Dr. Blackman, of Rome, Ga., but formerly of Tennessee, was also present. It was moved and carried that Dr. Blackman be elected an active member of the Association.

The next business being the election of officers, the Secretary read a letter of resignation from Dr. Scheibler, President of the Association, which after some discussion was accepted. Dr. Good was nominated for President and unanimously elected. This leaving the first vice-presidency vacant, Dr.

White was elected first Vice-President of the Association. These gentlemen, in neat speeches, thanked the members of the Association for the honor they had conferred on them, and promised to use their best endeavors to advance the interests of the Association. It was moved and carried that 300 copies of the Constitution and By-laws be printed, and that a number of copies be sent to each member throughout the State.

Dr. Rayen, Chairman of the Local Committee, moved that every member of the State Association should assist the Local Committee in the entertainment of the U. S. V. M. A., which was to convene in Nashville the next day. This was seconded and carried. The time and place of the next meeting was left to the Executive Committee, and, there being no other business, the meeting then adjourned.

JOSEPH PLASKETT, D.V.S.,
Secretary.

